# De-warping of images and improved eye tracking for the scanning laser ophthalmoscope

**DOI:** 10.1371/journal.pone.0174617

**Published:** 2017-04-03

**Authors:** Phillip Bedggood, Andrew Metha

**Affiliations:** Department of Optometry and Vision Sciences, University of Melbourne, Parkville, Victoria, Australia; Simon Fraser University, CANADA

## Abstract

A limitation of scanning laser ophthalmoscopy (SLO) is that eye movements during the capture of each frame distort the retinal image. Various sophisticated strategies have been devised to ensure that each acquired frame can be mapped quickly and accurately onto a chosen reference frame, but such methods are blind to distortions in the reference frame itself. Here we explore a method to address this limitation in software, and demonstrate its accuracy. We used high-speed (200 fps), high-resolution (~1 μm), flood-based imaging of the human retina with adaptive optics to obtain “ground truth” information on the retinal image and motion of the eye. This information was used to simulate SLO video sequences at 20 fps, allowing us to compare various methods for eye-motion recovery and subsequent minimization of intra-frame distortion. We show that a) a single frame can be near-perfectly recovered with perfect knowledge of intra-frame eye motion; b) eye motion at a given time point within a frame can be accurately recovered by tracking the same strip of tissue across many frames, due to the stochastic symmetry of fixational eye movements. This approach is similar to, and easily adapted from, previously suggested strip-registration approaches; c) quality of frame recovery decreases with amplitude of eye movements, however, the proposed method is affected less by this than other state-of-the-art methods and so offers even greater advantages when fixation is poor. The new method could easily be integrated into existing image processing software, and we provide an example implementation written in Matlab.

## Introduction

In image modalities that make use of point-focused raster scanning, each pixel in the reconstructed image is acquired at a different time. If the object of interest is in motion, this introduces distortion that cannot be removed *post hoc* unless a robust estimate of the object motion is available. In the case of retinal imaging especially, the constant and unavoidable motion of the fixating eye (tremors, slow drifts and microsaccades [1, 2]) compromises image fidelity. For current state-of-the-art methods such as scanning laser ophthalmoscopy (SLO) and optical coherence tomography (OCT), this issue imposes limitations on the ability to quantify fine differences in tissue structure [3], or track / target particular retinal features [4].

Current best-practice approaches in de-warping raster images of the retina come from adaptive optics scanning laser ophthalmoscopy (AOSLO), which seeks to image cellular detail and so demands high image fidelity. The most common method is to divide the frame to be recovered into many smaller regions of interest [5]. Since image information is acquired very rapidly in one direction (fast axis) and more slowly in the orthogonal direction (slow axis), these regions are rectangular "strips", with the long axis oriented in the fast direction of the scan. Each strip is then registered to some "key" reference frame. The key frame is chosen because it appears, subjectively or by some heuristic, to suffer from minimal distortion [3, 5]. Research efforts have been concentrated primarily on development of methods by which the anatomical tissue within each strip is correctly, and quickly, registered to the corresponding tissue in the reference frame [3, 5–7]. Such procedures allow each frame in an acquired sequence to be made to strongly match the reference frame, however, it is often lamented that inevitable distortions within the reference frame itself cannot be known or corrected [3, 6, 7]. Thus the identification (or generation) of a key frame that veridically represents the imaged anatomy is recognised to be of critical importance. Were fixational eye movements zero for a period of two or more frames, an excellent key frame could be chosen simply by identifying one of a pair of frames for which there is no relative distortion. However, given the statistics of normal fixational eye movements [1, 2], this situation is unlikely at current SLO scanning rates.

It was proposed some time ago that the distortion inherent in any reference frame can be minimized by registering all frames across a sequence, and averaging the results [6]. The idea is that the variations in position of the fixating eye are stochastic, and centered about a common point (the point of fixation). Therefore, given enough points of comparison, offsets in one direction should cancel with those in the other direction. Averaging the images themselves would produce a blurry final image due to the differences in distortion between each frame; instead, the shifts required for registration of each strip are averaged. This procedure is repeated across all strips in the reference frame, allowing the eye movements made during that frame to be recovered. This information can in turn be used to de-warp the frame [6].

In practice, the approaches of robust registration to a reference frame, and correction of the bias in that reference frame, can be combined [6], but the utility of doing so has not been explored. Our results here show that the combined utility is excellent and approaches ideal performance, and that the latter approach, which appears to have been largely forgotten, is in fact the more important of the two. Hence we have decomposed our analysis into the following 3 strip-based registration approaches:

a) "Key frame": Robust, conventional registration in which the corresponding anatomy is always located, if present in the imaged field. Since we are not concerned here with processing time, we achieved this by simply registering each strip to the full reference frame. De-warping of the reference frame is not attempted in this approach.

b) "Simplified de-warp": Removal of reference frame bias according to the average eye movement calculated for each strip. To isolate this aspect from the various strategies for robust registration that may be employed, no attempt was made to make the registration robust—strips are compared across frames if they have the same temporal offset relative to the beginning of their frame. This would lead to perfect overlap of anatomy in the case of zero eye movement, with loss of overlap and corresponding failure of registration if eye movements are too large. Although suggested some time ago [6], the importance of this method has not been determined and it appears to have received little attention in the field.

c) "Combined approach": The combination of the above two approaches, as first adopted by Vogel et al [6].

Since we have access to "ground truth" information as described below, we set out here to quantify the performance of the above approaches, and compare them with ideal performance.

## Methods

We acquired video sequences with high spatiotemporal resolution (200 fps, 1 μm) using a flood-illumination (non-raster) AO ophthalmoscope. Because each frame comprises pixels illuminated and captured simultaneously, distortions within frames are minimal. This "ground truth" data was used to simulate the acquisition of distorted AOSLO video sequences. We then trialed the various approaches outlined above to de-warp these images, and compared the output to our ground truth data.

### Acquisition of ground truth data

This project was carried out in accordance with the principles expressed in the Declaration of Helsinki, and approved by the University of Melbourne Human Ethics Committee. Written informed consent was obtained from the subject prior to testing.

Ground truth data was acquired from the cone photoreceptor mosaic of one human subject 1.25° temporal to the fovea. The subject (PB) was selected due to high optical quality and stability of fixation. The subject was positioned in a chin-and-forehead rest, and stared at a calibrated fixation grid while we used a flood-based adaptive optics ophthalmoscope, detailed previously [8], to image the retina.

The details of the adaptive optics system are summarized here as follows. The wavefront beacon is an 835-nm superluminescent diode (Hamamatsu, Hamamatsu City, Japan; power = 9.2 μW at the cornea). The wavefront sensor is a Hartmann-Shack with lenslets of 0.4-mm pitch and 24-mm focal length (Adaptive Optics Associates, Cambridge, MA), coupled to a charge-coupled device camera (Pike, Allied Vision Technologies, Stadtroda, Germany). The deformable mirror is a HiSpeed DM97-15 (Alpao, Montbonnot St. Martin, France) with 13.5 mm diameter, which corresponds to 7.6 mm in the pupil plane. The imaging light is generated by a broadband 8W supercontinuum laser pulsed at 80 MHz, and filtered through a SuperChrome tunable transmission filter (Fianium Ltd., Southampton, UK). Custom Matlab code was used to measure and correct the wavefront aberration of the eye at 30 fps before and during image acquisition. When RMS wavefront error reached < = 0.05 μm over a 7.0 mm pupil, an acquisition sequence of 5 s duration was triggered, with 750 ± 25 nm light (illuminating a 1.25° field on the retina, power = 0.67 mW at the cornea) captured at 200 fps with an Andor Neo sCMOS camera operating in global shutter mode (1000 frames of 2560 x 512 pixels, 2.5 ms exposure per frame, each pixel corresponds to ~0.48 μm on the retina).

**Fig 1:**
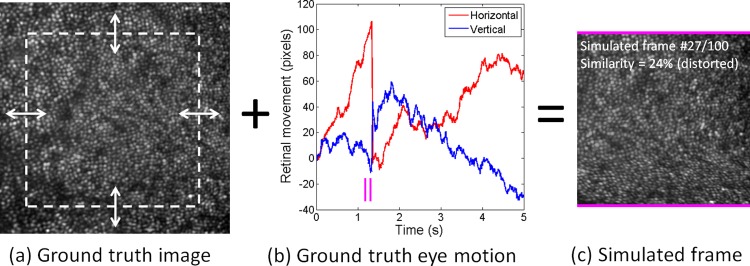
Ground truth data and generation of distorted SLO imagery. a) Cone photoreceptor mosaic 1.25° temporal to the fovea, generated from the average of 1000 frames acquired at 750 nm using flood-based adaptive optics. White box indicates the extent of the 400x400 pixel (192x192 μm) simulated SLO frame. b) Eye movement data in pixels (1 pixel = 0.48 μm) from the same sequence that was used to generate a. Eye motion was determined from cross-correlation of each of 1000 frames, measured at 200 fps, with the first frame in the sequence. c) One of the SLO frames simulated at 20 fps, generated by combining ground truth image and eye movement data (corresponding to time point indicated by magenta lines). Distortion is evident towards the bottom of the frame, which corresponds to a spike in eye movement. S1 Video shows all simulated frames for this sequence, after full-field registration of each frame to the first frame.

After image acquisition, images were background subtracted and flat-fielded to minimize any non-uniformities in the retinal illumination. We then applied full-field registration of all 1000 frames to the first frame in each sequence. Only translations were applied (rotation of the retinal scene was not observed). The 1000-frame average formed the “ground truth” image (shown in part in Fig 1A), while the x- and y-shifts required for registration formed the “ground truth” eye movement data (Fig 1B). We applied linear interpolation to the latter to generate quasi-eye movement data at 1000 fps, as opposed to our measured 200 fps.

### Simulation of SLO data

Using the ground truth retinal image and eye movement information, we simulated the acquisition of SLO data at 20 fps for 100 frames, i.e. for the same duration as our data acquisition. Individual rows in each frame were populated by indexing the ground truth image (Fig 1A) with an offset given by the ground truth eye movement information at the corresponding point in time (Fig 1B). We completed this procedure over a central 400x400 pixel window within the original ground truth frame, which gave enough redundant image area so that large eye movements did not render the SLO window empty (i.e. the ground truth image extends somewhat beyond the area shown in Fig 1A). Each pixel corresponded in size to ~0.48 μm on the retina, which is comparable to AOSLO devices [4]. An example generated frame is shown in Fig 1C, corresponding in time to the parallel magenta lines in Fig 1B.

For simplicity, our simulations assumed an SLO device operating at 100% duty cycle, with perfect de-sinusoiding such that distortions in the acquired SLO data arise only due to the motion of the eye and not from any device error. The simulation therefore represents an idealized SLO device.

We used a single averaged image (generated from 1000 sequentially acquired frames) to generate each SLO frame, meaning that we have not incorporated any realistic dynamics, other than eye motion, e.g. scintillation of photoreceptors, overlying blood flow, or shot noise. This is because we wished to assess the general validity of each approach without introducing temporal variations in image appearance. For similar reasons we focused on the photoreceptor mosaic and not the microvasculature.

### Recovery of eye movement and retinal image data

**Fig 2:**
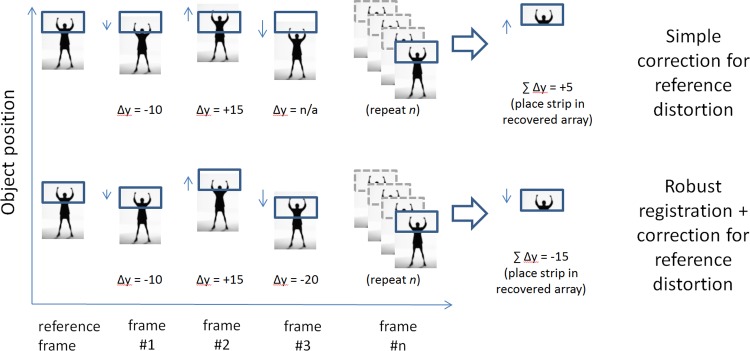
Depiction of two strategies to place rectangular strips in the correct position to minimize intra-frame distortion. Top: a simple procedure in which each strip in the reference frame is compared to the corresponding strip, relative to the beginning of the frame, in each other frame of the sequence. Here no special effort is made to locate the corresponding anatomy; when displacement becomes too great (frame #3), the displacement cannot be recovered or can be recovered only poorly, leading to a divergence between the methods for larger displacements. Bottom: As for top, but now a robust registration procedure is used which ensures that the corresponding anatomy is correctly located, if present in the imaged field.

Of the 3 approaches compared here, 2 attempt to de-warp the reference frame. These are illustrated in Fig 2. Our recommended approach to recover from motion-induced distortions within each frame is illustrated in Fig 2 (bottom), and is summarized as below. A simplified example has been made available for download in S1 Code.

1) Divide the frame to be recovered into *m* rectangular strips, with the long axis aligned with the fast axis of the scanner (blue rectangles in Fig 2, top).

- The height of each strip should not be too large (poor temporal fidelity of eye movement recovery) nor too small (poor quality of cross-correlation); we used a height of 31 pixels.
- Our strips were separated by 1 row, i.e. there was a high degree of overlap. Strips could be spaced some distance apart to improve computation time, but we did not explore this.

2) Register each strip to *n* other independently acquired full frames, where *n* is large (n = 100 in our simulations).

3) For each strip, average the *n* x-offsets, and *n* y-offsets, determined from step 2. This averaged set of measurements give a robust estimate of eye position during each frame.

4) For each strip, apply the offsets from step 3 to the central row of that strip, shifting its position within the recovery frame.

- We rounded displacements to the nearest pixel, which can cause either overlap in row placement (circumvented by averaging), or a lack of information in some rows (requiring 2D interpolation).

5) Use the recovered frame as a reference to which all other frames can be registered.

6) Optional: if image dynamics are not of interest (e.g. for determining cone location only), the above procedure can be applied to recover each frame in a sequence independently. All of these recovered frames can be averaged to produce a superior reference image. Another reason to recover all frames independently would be to validate that the method works (for example, the extent to which it is immune to natural variations in the pattern of eye movements, and hence distortions, encountered). Since all recovered frames are of the same tissue, they should appear undistorted with respect to one another. For the purposes of this paper, we therefore applied the above procedure to independently recover all frames in each simulated sequence and quantified their similarity to the true appearance of the tissue.

It should be noted that computation time on a modern PC for initial recovery of a frame is on the order of seconds (or minutes, if Step 6 is employed); the method is not suitable for real-time recovery of all frames. However, once an acceptable reference has been recovered then registration can easily proceed via more conventional, potentially real-time means [5].

The above procedure relies on the key assumption that there should be no systematic bias in the way that a particular piece of tissue becomes distorted across multiple SLO frames. That is, by viewing the same tissue many times and sampling from the full gamut of possible image distortions, we can become more and more confident of the true appearance of the tissue.

As mentioned earlier, the above procedure can be conceptualized as a combination of a) conventional robust registration methods, and b) the comparison of many registrations to determine precisely how the eye moved during acquisition of a particular frame, in order to de-warp that frame. To obtain a simplified implementation of the latter without interference from the former, one can modify Step 2, which would read:

2) Register each strip to *n* other strips, each acquired at the same temporal offset from the beginning of their respective frames.

A schematic of that procedure is shown for comparison, in Fig 2 (top). Note the scenario depicted for reference frame 3, where it is not possible to perform a meaningful cross-correlation due to lack of overlap of imaged tissue.

### Simulation of varying amplitudes of eye movement

As described above, our subject is well trained at maintaining steady fixation and so we expect the amplitude of eye movements in our simulated data to be relatively small. This was offset somewhat by choosing a relatively slow frame rate chosen for simulation (20 fps), which exacerbates intra-frame distortions caused by eye movements. It is known that the amplitude of fixational eye movements can vary quite markedly, especially in patients suffering from ocular disease [9]. To assess the impact of amplitude of eye movement on the various approaches explored here, we repeated the simulations for eye movements scaled on the range of 0.5 to 2.0 times the actual recorded eye movements.

### Quantification of performance

Fidelity of recovery was quantified by calculating the similarity (correlation coefficient) between the recovered SLO image and our ground truth flood image, using the largest common area between each image pair. Small differences in the size of the largest common area are not expected to significantly impact the results, due to the high spatial homogeneity of the cone photoreceptor mosaic. Recovered images were globally aligned (horizontally and vertically) with the ground truth image prior to calculating image similarity, as global image position is arbitrary.

We provide some example distorted imagery in the Results section, to provide some context as to the likely level of distortion corresponding to particular values of the correlation coefficient. For specific applications it may be more instructive to explore feature-based measures of image similarity; for example, calculating the apparent displacement of the centers of cone photoreceptors from their actual positions [3]. Such an approach was not used here, to avoid adding an additional layer of complexity in comparison of the approaches explored.

## Results and discussion

An inherent assumption of the methods explored here is that knowledge of eye movements during acquisition of each SLO frame can be used to completely recover that frame. To demonstrate the validity of this, we simulated an ideal case in which the ground truth eye movement data was already known, and each of the 100 simulated frames recovered. The RMS similarity metric in this case was >99.99%.

**Fig 3:**
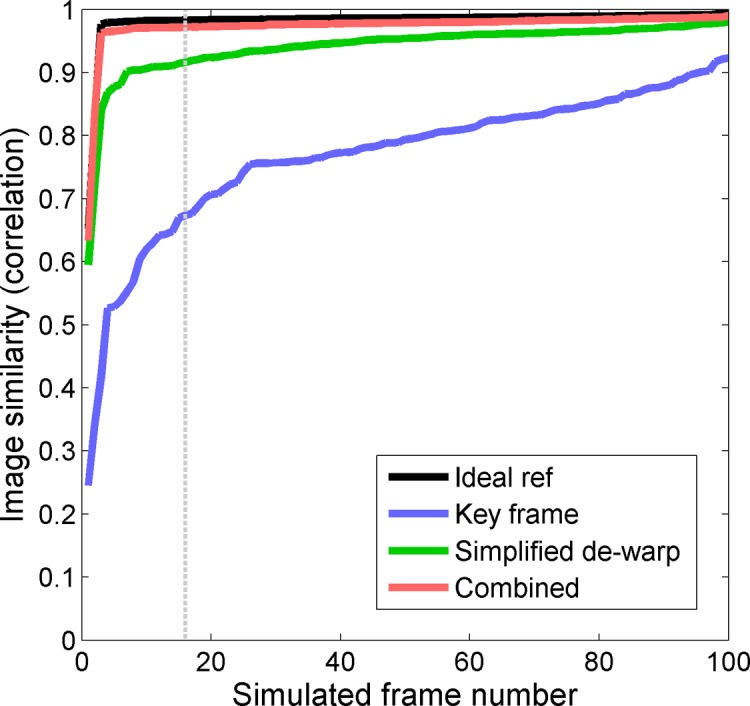
Image similarity to the ground truth image for 100 frames recovered from a sequence. Black: Ideal case in which the ground truth image is supplied as the reference for recovery, showing the upper limit of performance given the finite strip dimensions. Blue: Selection of a key frame, assuming perfect registration to that frame is achieved. Green: Simplified de-warp algorithm, in which eye movements made during the reference frame are determined and compensated by comparing strips acquired at the same time relative to the beginning of their frame (as described in Fig 2, top). Red: A combination of the above two methods. Grey line: Example frame compared for each method in S2 Video. Frames were re-ordered by increasing image similarity to aid visualization.

Accurate determination of intra-frame eye movement requires robust cross-correlation between strips. A trade-off emerges when choosing strip height, as taller strips increase robustness of cross-correlation in the case of whole-strip translation, but reduce it when eye movements are fast enough to produce intra-strip warp. To determine how well our chosen strip size approached ideal performance, we used an ideal reference frame, i.e. we supplied the ground truth image as the reference instead of recovering it by looping through 100 distorted frames. A plot of the resulting image similarity for our 100 recovered frames is shown in Fig 3 (black), and represents the upper limit of performance that we should expect to achieve. The similarity metric calculated in this way averaged 98.2 ± 3.7% across frames.

We now evaluate actual predicted performance where no ground truth information is given *a priori*. Fig 3 plots performance for three different methods as a function of the frames in one simulated sequence. The frames have been sorted in order of increasing similarity to the ground truth image. To gain better appreciation for the magnitude of distortion corresponding to different values of the image similarity metric, see the example frame recovered for each method in S2 Video.

Performance for the combined method (robust registration together with correction for reference frame bias) is plotted in red (mean = 97.4 ± 3.8%), the simplified correction for reference frame bias alone in green (mean = 94.1 ± 4.9%), and the most common approach in which a single key frame is selected in blue (mean of 76.9 ± 11.8%). The best single key frame to use from this sequence had an image similarity of 92.3% to the ground truth image (right-most point on blue plot). The combined method provided greater performance than the other methods, approaching the ideal performance indicated in black. The simplified correction for reference frame distortion, in which no attempts are made to improve the robustness of registration, can be seen to significantly outperform the single key frame approach.

When information on tissue dynamics is not required, fidelity of image recovery can be improved by recovering all *n* frames as opposed to a single reference frame, and averaging the results (Step 6 above). This increases processing time in proportion to *n*. Application of this step yielded a final similarity metric of 99.3% for the combined method, 98.6% for the simplified de-warp method, and 94.1% for the key frame method (the latter being simply the average of all potential reference frames, after full-frame registration).

**Fig 4:**
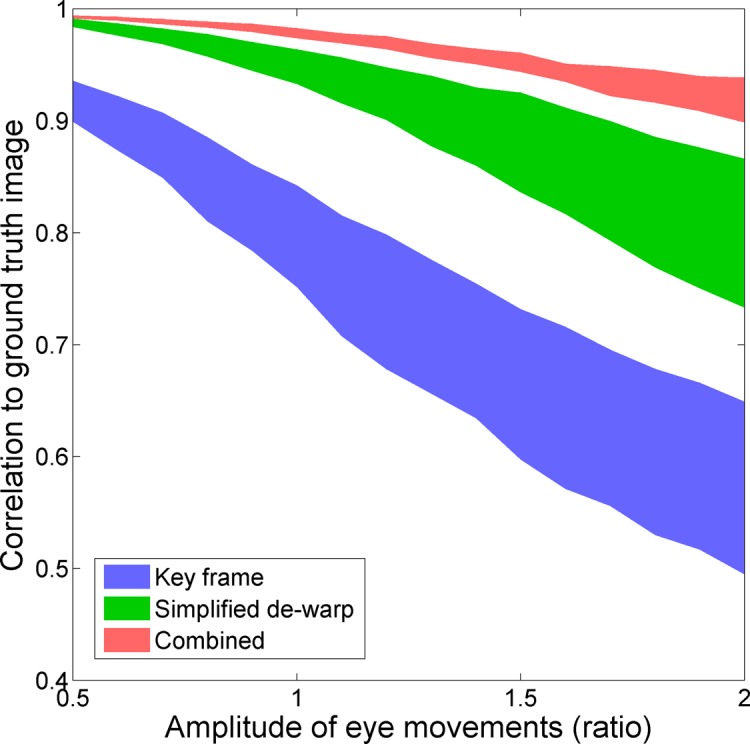
Performance as a function of increasing amplitude of fixational eye movements. Interquartile range (central 50% of data), for correlation of all recovered frames to the ground truth image, is plotted as a function of amplitude of eye movement.

Finally, we investigated the influence of amplitude of eye movements as described above. Results are shown in Fig 4, which plots image similarity interquartile range of 100 recovered frames, against eye movement amplitude ratio. The combined method (red) is more robust to increased amplitude of eye movements than either of the other methods (green, blue).

Nonetheless, the combined method was not immune to the influence of larger amplitude of eye movement, showing a clear decline. This occurred due to failure of registration, e.g. when the amplitude of eye movements was doubled from baseline, the maximum positional error for any strip compared with the ground truth rose from 1.4 μm to 8.2 μm (more than the semi-diameter of our strips which was 7.4 μm). In our simulation all imaged anatomy is always represented in the reference frame, and we register to the full reference frame; therefore failure of registration indicates that the tissue within the strip does not look sufficiently like the tissue in the reference frame for an accurate cross-correlation to be produced. In other words, we attribute the decline in accuracy with increasing amplitude of eye movement to non-negligible amounts of intra-strip distortion.

The amplitudes plotted in Fig 4 are relative to the baseline amplitude of fixational eye movements in this particular subject; an estimate of likely performance in other subjects could be made by scaling the results according to the absolute amplitude of the subject in question, compared to our subject. For example, the data included in S1 Code shows that in the case of unit amplitude, this subject had a peak-to-valley difference in vertical position of the eye of 50 μm over the course of the sequence, with a standard deviation of 12.4 μm.

## Conclusion

Fixational eye movements made during rasterized acquisition of retinal imagery can be recovered with high precision by image registration to many independently acquired frames. This information in turn allows accurate correction of intra-frame distortions that result from said eye movements. Such an approach has broad appeal since it can be implemented in existing devices, or be used to retroactively analyze existing data, without any change in hardware. Initial description of the approach by others did not receive widespread attention; we have revisited the general idea, showing that it approaches ideal performance in simulations. Our simulations did not take into account changes in retinal physiology, errors in de-sinusoiding, etc; therefore, confirmation of the benefits by comparison of actual raster scanned and flood image data obtained from the same eyes [3] is suggested.

## Supporting information

S1 Code Zip file containing an example Matlab implementation, together with sample data. (ZIP)

S1 Video Simulated SLO sequence. (MP4)

S2 Video Distortion compared across methods for an example recovered frame. (MP4)
