# Supplementary figures and images for: De-warping of images and improved eye tracking for the scanning laser ophthalmoscope
Source: PLoS One. 2017 Apr 3;12(4):e0174617. doi: 10.1371/journal.pone.0174617 (PMC5378343; doi:10.1371/journal.pone.0174617)

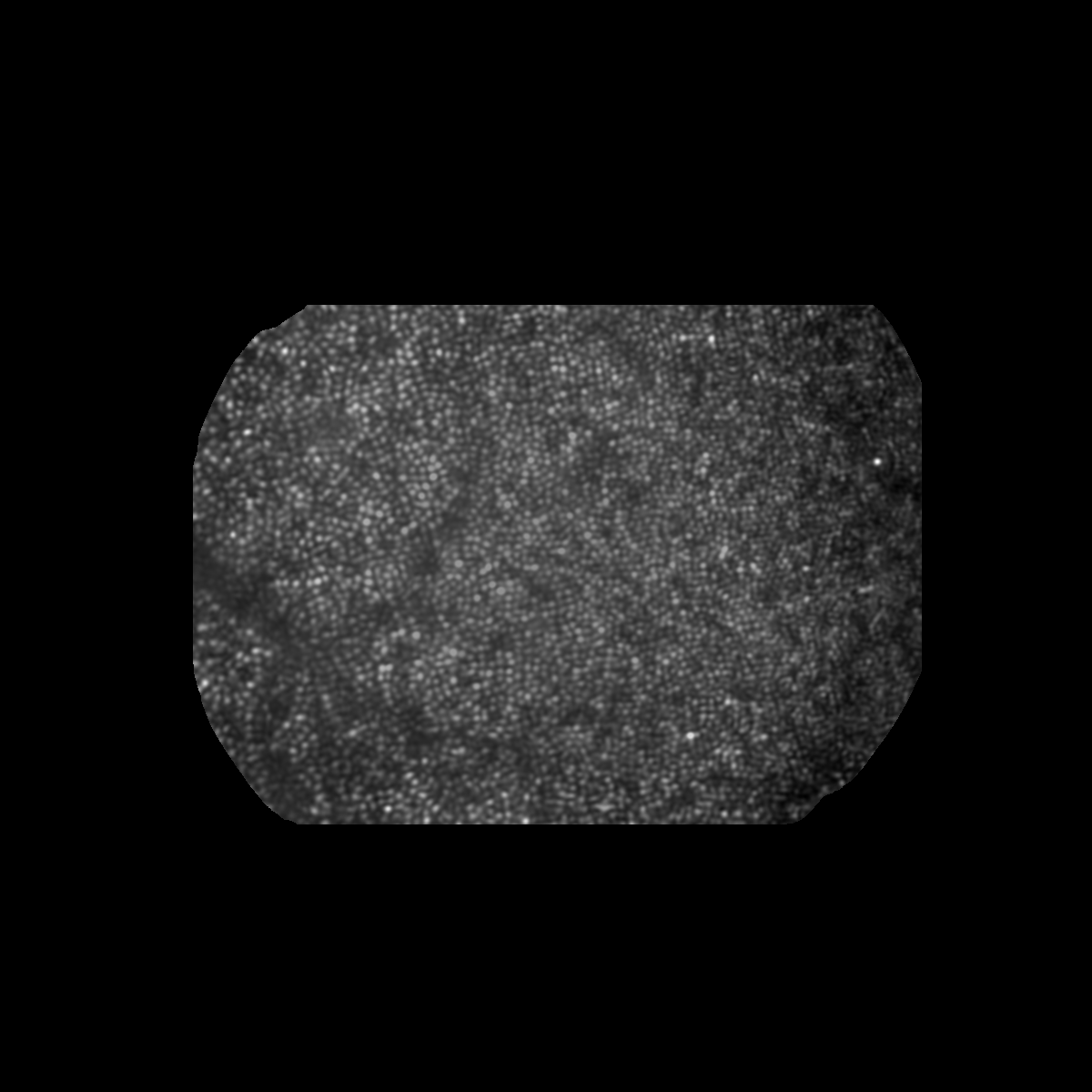

Supplement: S1 Code — (ZIP) [file pone.0174617.s001.zip › Ground truth image.tif]
